# Does Children’s Union Dissolution Hurt Elderly Parents? Linked Lives, Divorce and Mental Health in Europe

**DOI:** 10.1007/s10680-018-9501-5

**Published:** 2018-10-24

**Authors:** Marco Tosi, Marco Albertini

**Affiliations:** 1 Department of Social Policy, London School of Economics and Political Science, Houghton Street, London, WC2A 2AE UK; 2 Department of Political and Social Sciences, University of Bologna, Strada Maggiore 45, 40125 Bologna, Italy

**Keywords:** Divorce, Intergenerational relations, Mental health, Depression, Linked lives

## Abstract

Previous research has shown that parent’s union dissolution has negative consequences for individuals’ well-being, parent–child relationships and children’s outcomes. However, less attention has been devoted to the effects in the opposite direction, i.e. how children’s divorce affects parents’ well-being. We adopted a cross-country, longitudinal and multigenerational perspective to analyse whether children’s marital break-up is associated with changes in parents’ depressive symptoms. Using data from 17 countries and 5 waves of the Survey of Health, Ageing and Retirement in Europe (2004–2015), fixed effect linear regression models were estimated to account for time-constant social selection processes into divorce/separation. The results show that across European contexts parents’ depressive symptoms increased as one of their children divorced. Furthermore, we found that parents living in more traditional societies, such as Southern European ones, experienced higher increases in depression symptoms when a child divorced than those living in Nordic countries. Overall, the findings provide new evidence in support of both the notion of “linked lives” and a normative perspective of family life course events.

## Introduction

During the last decades separations and divorces have become increasingly common across European societies, with the crude divorce rate for the EU28 growing from 0.9 in 1970 to 2.0 in 2011 and 1.9 in 2012 and 2013 (divorces per 1000 inhabitants).^1^ The rising number of marital beak-ups has attracted scholars’ attention on the macro- and micro-level causes of divorce as well as on its socio-economic implications. Research on the consequences of divorce has consistently shown that marital dissolution is associated with a number of negative outcomes for the ex-partners. Divorce is associated with worse economic and housing conditions, particularly for women (Dewilde 2008; Feijten and Van Ham 2010; Uunk 2004); lower psychological well-being and health status (Amato 2000; Monden and Uunk 2013); and higher risks of social isolation and lack of informal care (de Jong Gierveld and Havens 2004; Pezzin and Schone 1999). Children are affected by parental divorce, too. They have higher risk of poverty (Backer 2015), lower educational achievement (Albertini and Dronkers 2009; Härkönen et al. 2017) and a higher probability of problematic behaviour in later life than children of married parents (Amato and Cheadle 2008; Strohschein 2005). The consequences of marital dissolution extend to intergenerational relations between adult–children and older parents. Previous studies have shown that parental divorce leads to a lower intensity of (non-resident) father–child contacts, poorer quality of parent–child relationship and a lower likelihood of receiving support from children in later life (Albertini and Garriga 2011; Kalmijn 2012, 2014; Tosi and Gähler 2016).

Most of the previous studies on the consequences of marital dissolution, however, have paid relatively little attention on the impact that children’s divorce or separation can have on the well-being of their parents. The existing evidence on such intergenerational effects indicates that children’s marital break-up may have either no or negative effects on parents’ mental health (Kalmijn and De Graaf 2012; Milkie et al. 2008). The aim of the present paper is to analyse this relation and thus explore whether children’s divorce is associated with an increase in older parents’ depression symptoms in different European contexts. Unlike previous studies, we estimate fixed effects panel models to account for possible selection bias due to time-constant individuals’ and families’ characteristics. In addition, the analysis addresses the role of potentially important moderating factors: the presence of young grandchildren and the social context in which divorce takes place. We explore how the association between children’s divorce and parents’ mental well-being varies across different European contexts characterized by different family orientations. The impact of children’s divorce on parents’ mental health may vary considerably depending on the level of social acceptance towards divorce in a given cultural and institutional setting.

## Parents’ Mental Health and Children’s Divorce

Depression is one of the most serious health problems in later life and has important implications for individual’s quality of life (Blazer 2003). Previous studies have shown that the number of children and the quality and intensity of family relationships play a pivotal role in preventing older parents’ depression (Bengtson et al. 2002; Grundy et al. 2017). Thus, for example, having frequent meetings with adult children significantly reduces the number of parents’ depression symptoms (Teo et al. 2015). Similarly, previous studies indicate that living with a child has a beneficial effect on parents’ mental health (Aranda 2015; Courtin and Avendano 2016), although the meaning of intergenerational co-residence varies dramatically across contexts and a negative effect of returning home by adult–children has been found in a group of Nordic countries (Tosi and Grundy 2018b).

While the effect of intergenerational relations on parents’ well-being has been extensively documented in previous research, there are only few studies that explore the extent to which children’s life course events may have a direct effect on parents’ mental health. Parents and children constitute a latent network of support, a convoy of significant others who protect and socialize each other throughout the life course (Antonucci and Akiyama 1987; Antonucci et al. 2011; Litwin 2009). Family members live “linked lives”, and their respective life courses mutually influence the well-being of other family generations (Elder 1994). In particular, there are various social mechanisms through which children’s divorce/separation may (negatively) affect parents’ mental health: (1) parents’ empathy with the child’s status when the latter is undergoing a marital dissolution; (2) feelings of responsibility towards children’s life course choices; (3) parents’ frustration due to the child’s failure to meet parents’ expectations about the ideal life course of their offspring, (4) changes in the intensity and quality of parent–child relations. In addition, a number of factors may moderate these mechanisms; two of the most prominent are the presence of young grandchildren and the social norms characterizing the specific social context in which the family lives.

First, parent–child relationships are usually characterized by intimacy and empathy. Older parents develop feelings of empathy towards their adult–children’s lives, and thus both positive and negative events in children’s life course can reverberate into the mental health status of the parents (Batson 1998; Lawton et al. 1994; Knoester 2003). Studies show that children’s exposure to problematic and stressful experiences, such as illnesses, financial hardships, and drinking or drug problems, is associated with a decline in parent’s mental health (Greenfield and Marks 2006; Milkie et al. 2008; Pillemer and Suitor 1991; Pillemer et al. 2017). When divorce leads to the interruption of a very conflictive relationship between partners, marital break-up can have no effect on well-being or even imply some psychological benefits for both adult children and their parents. Using OLS change-score models, Milkie et al. (2008) found no associations between adult children’s divorce and changes in parents’ depressive symptoms in a sample of American families. Conversely, when divorce is experienced as a shock and a stressful experience by both adult children and their older parents, it may lead to an increase in the number of depressive symptoms and mental health problems of both family generations. In a longitudinal study on the Netherlands, Kalmijn and De Graaf (2012) used OLS change-score models and showed that children’s marital break-up was associated with increases in the depression score of parents, and such effect was larger for parents with traditional family attitudes.

Second, parental feelings of responsibility towards their children’s lives might also be a way through which children’s divorce affects parental mental health in later life. Parents have socialized their children during childhood and adolescence and thus may feel partly responsible for their children’s life course choices. Hagestad (1985) has shown that American mothers perceived offspring’s difficulties in becoming independent as a personal failure in their socialization role. In a similar vein, parents may experience feelings of failure and disappointment when adult children fail to maintain their marital relationship.

Third, parents develop expectations about their children’s life courses. Previous research has shown that when children do not meet these expectations, parents experience stress and feelings of failure for parents (Aquilino and Supple 1991; Pillemer et al. 2012). A child’s marital break-up can be perceived as a failure vis-à-vis parents’ expectations, a deviation from a normative life course and, ultimately, the failure “to keep up with the Joneses’ children life course”. Children’s failure to maintaining the marriage/partnership status may lead parents to experience a sense of guilt, shame and failure, together with ambivalent feelings of conflict and solidarity (Sechrist et al. 2011; Pillemer et al. 2010; Pillemer and Suitor 2002; Orth et al. 2009). These emotional states may have important implications for the mental health of older parents, especially when they hold traditional family attitudes and strongly support the institution of marriage (Kalmijn and De Graaf 2012).

Fourth, parent–child relations, their intensity and quality, are often affected by individual’s life course transitions. When children exit the parental home, enter into a relationship, get married or become parents, the frequency, type and quality of contacts as well as the intensity and direction of support exchange are likely to change (Albertini 2016; Bucx et al. 2008; Sarkisian and Gerstel 2008; Ward et al. 2014). Divorce is no exception to this. Previous studies have shown that while parental divorce leads to a reduction in parent–child contact, children’s divorce elicits parental support and increases the likelihood of having frequent contact or intergenerational (re)co-residence (Albertini et al. 2018; Guzzo 2016; South and Lei 2015; Stone et al. 2014; Tosi and Grundy 2018a). The increase in the intensity of intergenerational relations following a child’s marital break-up may have ambiguous and opposite effects on the well-being of parents. On the one hand, parent–child contact frequency has positive consequences on parents’ mental health (Bengtson and Roberts 1991; Buber and Engelhardt 2008; Tosi and Grundy 2018b). Thus, for instance, Teo et al. (2015) have shown that in the USA, after controlling for parent–child relationship quality, having weekly or more face-to-face contact with children decreases the likelihood of developing depression symptoms in later life. On the other hand, the emotional stress connected with marital dissolution can be transmitted from children to parents via their social interactions—a mechanism known as mood contagion (Wethington 2000; Wolff et al. 2015). Moreover, the burden of providing intense support to divorced children may lead to a deterioration in the quality of relationships between family generations and intergenerational conflict (Pillemer et al. 2010; Timonen et al. 2011; Kaufman and Uhlenberg 1998). Conflicting or stressful relationships with children—or relations that are perceived as strongly imbalanced in terms of support given or received—may have a detrimental effect on parents’ well-being (Djundeva et al. 2015; Koropeckyj-Cox 2002; Silverstein et al. 1996; Ward 2008).

In light of the social mechanisms and research findings mentioned above, we expect that a child’s divorce is associated with increases in parents’ depression symptoms (Hypothesis 1). We also test whether the negative effect of children’s marital break-up varies depending on the gender of the parent. Because of the matrifocal nature of kin relations and the role of women as kin keepers in family systems (Rossi and Rossi 1990), children’s life choices may have a larger effect for mothers than for fathers. Mothers invest more in family relationships, feel deeply involved in their offspring’s lives and may suffer more from a child’s marital break-up.

Furthermore, the effect of children’s divorce on parents’ mental health can be moderated by the presence of young grandchildren. The social stigmatization of divorce tends to be stronger when small children are involved, because parents are expected to live together to socialize their children during childhood and adolescence (Liefbroer and Billari 2010). In addition, grandparents’ depression score may increase as a result of the loss of contact with grandchildren after parental divorce (Drew and Silverstein 2007). Given that marital break-up in the middle generation is negatively associated with the intensity and quality of grandparent–grandchild relations, particularly in paternal lineage (Albertini and Tosi 2018; Jappens and van Bavel 2016; Westphal et al. 2015; Silverstein et al. 2003), grandparents may suffer from their children’s divorce. Consequently, we hypothesize that the presence of a young grandchild exacerbates the negative consequences of children’s divorce on parents’ depressive symptoms, especially for paternal grandparents (Hypothesis 2).

### Family Dissolution in the European Context

Values and social norms are important factors moderating the potential effect of a child’s divorce on elderly parents’ mental well-being. At the micro-level, Kalmijn and De Graaf (2012) found that the effect of a marital break-up on parental mental health varies according to parents’ family values, and it is stronger for those holding more traditional family norms. At the macro-level social norms characterizing European contexts may have a role in shaping the meaning and effect of a child’s union dissolution (Liefbroer and Billari 2010; Lyngstad 2011). Previous studies of the consequences of marital dissolution have suggested that the negative effects of divorce decrease with the increasing frequency and social acceptance of marital dissolution across social contexts. In other words, the declining effect hypothesis predicts that the intensity of the negative effects of divorce is inversely related to the amount of social stigma associated with marital dissolution and with the frequency of divorces in a specific society (Albertini and Garriga 2011). The evidence on this hypothesis, though, is mixed. Depending on the methods adopted and the specific outcome of interest, previous research findings either support ([social contact] Kalmijn and Uunk 2007; [well-being] Sigle-Rushton et al. 2005; Verbakel 2012) or reject the hypothesis ([parent–child contact] Albertini and Garriga 2011; Tomassini et al. 2004; [children’s education] Kreidl et al. 2017).

Social norms and the acceptance of divorce may vary considerably across countries, together with the diffusion of the event, divorce legislation, gender roles, as well as family orientations and religious traditions (Surkyn and Lesthaeghe 2004). Within Europe, divorce and separation rates are the highest in Nordic countries, whereas they are the lowest in Mediterranean and Catholic societies where familialistic attitudes are prevalent and social disapproval of divorce is the highest. Continental and Eastern European countries fall in between. Clearly, there is also considerable heterogeneity within these macro-geographical areas and even within each country (Jappens and Van Bavel 2012, 2016; Kalmijn 2010; Kalmijn and Uunk 2007).^2^ Thus, for instance, among Eastern countries Poland is characterized by strong religious traditions and a prominent role of the Catholic Church in the society (Rijken and Liefbroer 2012).

Due to sample size limitations, in the present paper we are not able to address variations in the different relevant macro-level dimensions associated with the effect of children’s divorce. Thus, we analyse differences across four groups of countries (Nordic, Continental, Eastern and Southern) that—despite characterized by significant internal heterogeneity—broadly identify European contexts with similar levels of diffusion and social acceptance of marital dissolution. Older parents in family-oriented societies, such as Southern European ones, tend to have more conservative attitudes against divorce and may expect that their children maintain their marital relationships throughout the life course. Studies indicate that parents in Mediterranean countries suffer from socially unaccepted behaviour by adult children and try to discourage them in adopting “new” family behaviours using material and psychological sanctions (Di Giulio and Rosina 2007; Rosina and Fraboni 2004). Conversely, older parents’ mental health may be less affected by their adult children’s life course choices in some Western and Nordic societies, where parents have less conservative and traditional values. We hypothesize, therefore, that the negative association between children’s divorce and parents’ mental health is smaller in Nordic than in Southern and Eastern European countries where divorce is less common and more socially stigmatized (Hypothesis 3).

## Data and Methods

### Sample

The data used in this study come from the *Survey of Health, Ageing and Retirement in Europe* (SHARE) that is a cross-national, longitudinal survey representative of the ageing population in Europe. In the first five regular waves—which took place in 2004/2005, 2006/2007, 2011, 2013 and 2015—the survey gathered information on individuals aged 50 years or older and their partners.^3^ SHARE also collects detailed information on up to four selected children of each respondent. We adopted the parent–child dyad as the unit of analysis and used the reported sex and date of birth of each child to link children’s information across waves.

The analysis was performed on parent–child dyads in which children were aged 18–55 at baseline.^4^ Dyads including children above 55 years were excluded because union dissolution at older ages is quite exceptional in European countries. Adult children who were already divorced/separated at the baseline or lived without a partner throughout the entire observation period were excluded from the sample, as they were not at risk of divorce (47%). We also restricted the sample to parents who were born in the country of residence as foreigners may have different and heterogeneous cultural orientations towards marital break-up (5.3%). Respondents who lacked information on variables of interest (3.6%) and those who were present in only one wave were excluded as the analysis focuses on changes in depressive symptoms over time. Israel—the only non-European country in the data—was not considered in the analysis. The final sample includes 35,685 parents, 59,094 parent–child dyads, and 160,732 observations for 17 countries: Sweden, Denmark, the Netherlands, Austria, Germany, Switzerland, Luxemburg, France, Belgium, Slovenia, Spain, Portugal, Italy, Greece, Czech Republic, Poland and Estonia.

### Dependent Variable

The dependent variable was measured through the EURO-D depression scale that is based on 12 items—i.e. depression, pessimism, suicidality, guilt, sleep, interest, irritability, appetite, fatigue, concentration, enjoyment and tearfulness—and ranges from 0 to 12. The scale’s internal consistency has been tested and confirmed in previous research (e.g. Prince et al. 1999; Ploubidis and Grundy 2009). The distribution of depressive symptoms is typically skewed and includes an excess of zeros. Under these circumstances, OLS assumptions tend to be violated. However, errors’ distribution can be approximate (at 95% level) to a normal distribution when, instead of focusing on EURO-D score, we analyse changes in depression score over time. Diagnostic analyses performed on our fixed effects estimates showed that the normality and independence assumptions were not violated.

### Independent Variables

The main independent variable utilized in the analysis was derived from a question about children’s marital status. The six original answer categories are as follows: (1) married and living together with spouse, (2) registered partnership, (3) married, living separated from spouse, (4) never married, (5) divorced and (6) widowed. Divorce and separation of marriages were treated as equals, whereas it was not possible to identify the dissolution of de facto partnerships. The variable regarding changes in a child’s marital status from one wave to the next was dichotomized distinguishing between adult children who stayed in partnership (categories 1 and 2) and those who became divorced or separated across waves (categories 3 and 5). Those children who divorced during the observation period were then excluded from the sample in the subsequent waves (right censoring) to analyse the short-term effect of the transition from partnership to divorce. This strategy also allows us to exclude reverse transitions from divorce to re-partnership. We observed 1314 and 1765 transitions to divorce in father–child and mother–child dyads, respectively (see Table 1).

**Table 1:** Sample characteristics

	Fathers		Mothers		Changes	
	% or mean	*N*	% or mean	*N*	In	Out
Dependent variable						
Parents’ depressive symptoms	1.9 (1.9)		2.7 (2.3)		0.1 (2.1)	
Children’s characteristics						
Transition to divorce	2.0	1314	1.9	1765	2.0	0.0
Child birth	78.2	52,418	80.5	75,418	12.4	0.0
Employment status						
Employed	85.9	57,573	85.4	80,034	36.5	5.7
Unemployed	3.5	2353	3.7	3426	2.3	58.9
Student	1.0	698	0.9	845	0.3	70.1
Not in LM	9.6	6410	10.0	9393	3.9	37.4
Parents’ characteristics						
Age (*sd*)	68.9 (8.6)		67.5 (8.9)		2.8(1.5)	
Marital status						
Partnered	86.0	57,639	65.8	61,645	0.7	2.8
Separated	6.7	4486	10.2	9552	0.2	2.4
Widowed	7.3	4909	24.0	22,501	2.5	0.3
Employment status						
Employed	17.5	11,739	15.4	14,452	1.5	35.2
Retired	76.5	51,267	58.3	54,610	28.1	5.2
Unemployed/not in LM	6.0	4028	26.3	24,636	5.2	32.9
No. of chronic diseases (*sd*)	1.7 (1.5)		1.9 (1.6)		0.1 (1.4)	
No. of mobility limitations (*sd*)	1.3 (2.0)		2.0 (2.4)		0.2 (1.9)	
At least 1 limitation in IADLs	13.1	8761	19.7	18,499	12.0	43.2
At least 1 limitation in ADLs	10.3	6884	10.9	10,224	7.5	48.9
Contact frequency						
Weekly or more	78.4	52,547	80.2	75,124	36.5	10.8
Less than weekly	19.4	13,036	17.5	16,439	10.3	39.2
Co-residence	2.2	1451	2.3	2135	0.4	23.6
Child at baseline						
Child > 14	16.4	10,977	20.7	19,453	–	–
No children	25.3	16,610	22.5	21,068	–	–
Child ≤ 14	58.3	39,061	56.7	53,177	–	–
Country groups					–	–
Nordic	21.6	14,291	20.0	18,763	–	–
Continental	42.0	28,187	41.8	39,107	–	–
Southern	19.3	12,916	19.3	18,120	–	–
Eastern	17.1	11,440	18.9	17,708	–	–
No. of observations	100.0	67,034	100.0	93,698		
No. of dyads	100.0	25,030	100.0	34,064		
No. of parents	100.0	15,080	100.0	20,605		

The time span occurring between consecutive waves varies remarkably according to different waves and countries. Multiple transitions may occur between two consecutive waves, and for example, never married children might become separated in the later wave. Two sensitivity analyses were performed to account for differences in time spans between waves. First, we utilized an independent variable distinguishing between transitions to divorce or separation that occur within a 2-year interval and in a longer time interval. Second, we included in the sample not only children who were partnered, but also those who were un-partnered, to identify children’s transitions from never married or partnered to divorce or separation. In both cases the results obtained were in line with those presented in the text.

A relevant control variable used in the analysis is contact frequency between parents and children. As mentioned in the theoretical background, an increase in contact frequency may be a channel through which negative life course events, such as divorce, reverberate in parental mental health. Contact frequency was measured as the total number of meetings or other interactions (e.g. phone calls or e-mails) during the 12 months previous to the interview and was coded into two categories: weekly or more and less than weekly. Consequently, in the fixed effects models the former category indicates an intensification of parent–child contacts, whereas the latter means that there was a decrease in contact frequency from one wave to the next. A further category was added to account for intergenerational co-residence, which we can consider as an intensification of parent–child relations.

Children’s own parenthood status was captured by a time-varying variable concerning whether a child was born or not between two consecutive waves. In the second step of our analyses, we distinguished between adult children having a young child aged 0–14, those having a child aged 15 or over and those without children at the baseline. The 14 years cut point was chosen on the basis of previous research findings on grandparenting role (Aassve et al. 2012). The categorical variable allows us to test whether the association between the transition to divorce or separation and parents’ depressive symptoms was moderated by the presence of a young grandchild.^5^

Other covariates used in the multivariate analysis refer to parents’ and children’s socio-demographic characteristics. Children’s employment status was grouped into four categories: employed, unemployed, student, and not in the labour market. Parents’ characteristics included in the analysis were as follows: age, age-squared, marital status (partnered, separated and widowed) and four different indicators of physical health: the number of chronic diseases that parents reported during the interview included (range from 0 to 10 or more); the number of reported mobility limitations (range from 0 to 10 or more); and finally, we included two dummy variables identifying parents who had at least one limitation in activities of daily living (ADLs) or instrumental activities of daily living (IADLs). The former refers to needing help from others to perform tasks such as bathing, dressing or eating, while the latter indicates difficulties in activities such as preparing a hot meal, shopping for groceries or making telephone calls.

As mentioned in the theoretical background, the different European countries were grouped into four clusters: Southern (Italy, Greece, Spain and Portugal), Eastern (Czech Republic, Poland and Estonia), Continental (Austria, Germany, France, Switzerland, Belgium, Luxemburg and Slovenia) and Nordic (Sweden, Denmark and the Netherlands) European countries. A number of sensitivity analyses were performed using alternative country classifications and excluding, for example, the Netherlands, Estonia and Poland from the sample. The results show a consistent north–south divide in the effect of children’s divorce on parents’ mental health.

### Analytical Strategy

We used fixed effects linear regression models to examine the extent to which adult children’s transition to divorce was associated with *changes* in parents’ depressive symptoms. Fixed effects models allow us to analyse concomitant changes in children’s partnership and parent’s mental health symptoms excluding the influence of time-constant characteristics. As a matter of fact, a number of unobserved factors may affect both adult children’s propensity to divorce and the occurrence of depressive symptoms in parents, and thus introduce a bias in the estimates of a conventional regression analysis. For example, parental conflicts and a negative environment in early childhood may prompt adult children to divorce or separate (e.g. Amato and DeBoer 2001) and may be carried over into parents’ depressive symptoms in later life (Kalmijn and Monden 2006). Our modelling strategy has the advantage to account for these possible sources of bias as time-fixed individual and household effects.

The analytical strategy implemented consists of three steps. Firstly, the association between children’s union dissolution and changes in parents’ depressive symptoms was analysed in the overall sample as well as for the subsamples of elderly fathers and mothers separately; thus, the analysis accounts for potential differences in the effect of child’s divorce by parent’s gender. Since the results for fathers and mother were substantially equivalent, in the second step of the analysis we did not further distinguish our subsamples by parent’s gender. The second step aims at examining the moderating role of the presence of young grandchildren among maternal and paternal grandparents. The analysis was performed for adult sons and daughters separately, because maternal and paternal grandparents are likely to have different relationships with their grandchildren after divorce or separation (Westphal et al. 2015). In the two subsamples we added the interaction between children’s divorce/separation and a dummy for having a child aged 0–14 at baseline to test whether the effect of union dissolution was larger when a young child was involved. The third step consists of four regression models fitted for each country group separately. As a further test of the variation of the effect of child’s divorce across the different country clusters, we included interaction terms between the transition to divorce and the four country clusters in the overall regression model. These latter analyses were performed on the pooled sample of mothers and fathers, given the limited number of transitions within each specific cluster (787 in Nordic countries; 1366 in Continental ones; 400 in Southern Europe; 528 in Eastern countries).

## Results

Table 2 reports the results of the first step of our analysis; the results from fixed regression models indicate that there was an association between children’s transition to divorce and the number of parents’ depressive symptoms. In line with the first hypothesis, the number of depressive symptoms of fathers and mothers increased when one of their adult children became divorced. This association held after controlling for children’s transitions into unemployment and parenthood; moreover, its size did not differ significantly between fathers (Coef. = 0.10; *p* value < 0.05) and mothers (0.14; *p* value < 0.01), suggesting that there were no relevant differences by parent’s gender. These differences were further tested by fitting the model on the overall sample and including an interaction term between parent’s gender and child transition to divorce; the interaction coefficient was not significant.

**Table 2:** Fixed effects linear regression models on the number of parents’ depressive symptoms

	Overall		Fathers		Mothers	
	Coef.	SE	Coef.	SE	Coef.	SE
Children’s characteristics						
Transition to divorce	0.13**	(0.04)	0.10*	(0.05)	0.14**	(0.05)
Childbirth	0.02	(0.04)	− 0.01	(0.05)	0.05	(0.05)
Employment status (*Ref.* employed)						
Unemployed	0.12**	(0.04)	0.15**	(0.06)	0.10*	(0.05)
Student	0.05	(0.06)	0.04	(0.07)	0.06	(0.08)
Not in LM	0.02	(0.03)	0.01	(0.04)	0.03	(0.04)
Parents’ characteristics						
Age	− 0.24**	(0.03)	− 0.17**	(0.04)	− 0.28**	(0.04)
Age^2^	0.01**	(0.00)	0.01**	(0.00)	0.01**	(0.00)
Marital status (*Ref.* married)						
Divorce or separated	− 0.09	(0.16)	− 0.37	(0.25)	0.09	(0.20)
Widowed	0.58**	(0.08)	0.88**	(0.15)	0.49**	(0.09)
Employment status (*Ref.* employed)						
Retired	− 0.04	(0.03)	− 0.06	(0.04)	− 0.03	(0.05)
Unemployed/not in LM	0.10*	(0.04)	0.12+	(0.06)	0.08	(0.05)
No. of chronic diseases	0.12**	(0.01)	0.11**	(0.01)	0.12**	(0.01)
No. of mobility limitations	0.17**	(0.01)	0.18**	(0.01)	0.16**	(0.01)
At least one limitation in IADLs	0.30**	(0.03)	0.37**	(0.05)	0.27**	(0.03)
At least one limitation in ADLs	0.35**	(0.04)	0.41**	(0.05)	0.31**	(0.05)
Parent–child contact frequency (*Ref.* weekly or more)						
Less than weekly	0.05*	(0.02)	0.04	(0.03)	0.05*	(0.03)
Co-residence	0.03	(0.07)	− 0.06	(0.09)	0.11	(0.10)
Constant	11.67**	(1.31)	7.45**	(1.92)	14.09**	(1.76)
R-squared	0.07		0.09		0.06	
No. of parents	35,685		15,080		20,605	
No. of dyads	59,094		25,030		34,064	
Observations	160,732		67,034		93,698	

Standard errors are clustered for parents. Control variables include dummies for waves, not reported in the table

**p < 0.01, *p < 0.05, +*p* < 0.1

It is worth noting, however, that increases in parents’ depressive symptoms associated with a child’s union dissolution were relatively small. Other factors, such as becoming widowed (Coef. = 0.88 for fathers and 0.49 for mothers) and developing limitation(s) in daily activities (Coef. = 0.41 for fathers and 0.32 for mothers), had larger effects on parents’ depressive symptoms.^6^ On the other hand, the size of the coefficients suggests that parent’s depressive symptoms increased as a child separated to a similar extent as becoming unemployed (Coef. = 0.15 for fathers and 0.10 for mothers), indicating that intergenerational effects from children’s marital break-up to parents’ mental health were relevant.

Parent–child contact frequency was included as a possible mediator in the association between child’s transition to divorce and changes in parent’s depressive symptoms. In line with previous research findings (Teo et al. 2015), decreasing contact with a child after his/her divorce—i.e. from more weekly to less than weekly—was associated with increases in parents’ depressive symptoms, whereas the coefficient related to living with parents (intergenerational co-residence) does not reach standard levels of statistical significance. Further analyses confirmed that the relation between union dissolution and parents’ depressive symptoms was not mediated by changes in parent–child contact frequency and living arrangements.^7^

### Child’s Divorce, Grandchildren, and (Grand)Parents’ Mental Health

The models reported in Table 3 test the hypothesis that the positive association between children’s divorce and parents’ depressive symptoms was stronger when a young grandchild was involved. Since previous studies have indicated that family break-up has a different effect on paternal and maternal grandparents, the regression model was fitted for sons and daughters separately.^8^ The results show that parents’ depressive symptoms increased as their sons (Coef. = 0.09; *p* value < 0.05) and daughters (Coef. = 0.16; *p* value < 0.05) became divorced or separated. Among sons, the interaction between divorce and the presence of a young (grand)child was not statistically significant, though the related coefficient was in the expected direction. The interaction effect was positive but still not significant also in the case of childless sons. Among daughters there were no significant differences between the effect of the divorce of those with a child aged 0–14 and those with an older child. On the other hand, the regression results suggest that there was no effect of child’s divorce on parent’s mental health if the union dissolution involved a childless daughter, whereas this association was significant among daughters with children. The presence of a (grand)child, independent of their age, seems to be a necessary condition to produce a negative effect of the daughter’s divorce on parent’s mental health. Overall, these results indicate that the hypothesis that the presence of a young grandchild exacerbates the negative effect of a child’s divorce on grandparents’ mental health should be rejected in the case of both maternal and paternal grandparents.

**Table 3:** Fixed effects linear regression models on the number of parents’ depressive symptoms

	Sons				Daughters			
	Coef.	SE	Coef.	SE	Coef.	SE	Coef.	SE
Children’s characteristics								
Transition to divorce	0.09*	(0.04)	− 0.05	(0.13)	0.16**	(0.05)	0.32**	(0.11)
Childbirth	− 0.01	(0.05)	− 0.01	(0.05)	0.06	(0.05)	0.06	(0.05)
Employment status (*Ref.* employed)								
Unemployed	0.06	(0.06)	0.06	(0.06)	0.14**	(0.05)	0.14**	(0.05)
Student	0.01	(0.09)	0.01	(0.09)	0.07	(0.07)	0.07	(0.07)
Not in LM	0.05	(0.07)	0.05	(0.07)	0.01	(0.03)	0.01	(0.03)
Parents’ characteristics								
Age	− 0.23**	(0.03)	− 0.23**	(0.03)	− 0.25**	(0.03)	− 0.25**	(0.03)
Age^2^	0.00**	(0.00)	0.00**	(0.00)	0.00**	(0.00)	0.00**	(0.00)
Marital status (*Ref.* married)								
Divorce or separated	− 0.38+	(0.21)	− 0.38+	(0.21)	0.15	(0.18)	0.15	(0.18)
Widowed	0.48**	(0.09)	0.48**	(0.09)	0.68**	(0.09)	0.68**	(0.09)
Employment status (*Ref.* employed)								
Retired	− 0.01	(0.04)	− 0.01	(0.04)	− 0.05	(0.04)	− 0.05	(0.04)
Unemployed/not in LM	0.13*	(0.05)	0.13*	(0.05)	0.07	(0.05)	0.07	(0.05)
No. of chronic diseases	0.12**	(0.01)	0.12**	(0.01)	0.11**	(0.01)	0.11**	(0.01)
No. of mobility limitations	0.16**	(0.01)	0.16**	(0.01)	0.17**	(0.01)	0.17**	(0.01)
At least one limitation in IADLs	0.29**	(0.03)	0.29**	(0.03)	0.31**	(0.03)	0.31**	(0.03)
At least one limitation in ADLs	0.33**	(0.04)	0.33**	(0.04)	0.38**	(0.04)	0.38**	(0.04)
Parent–child contact frequency (*Ref.* weekly or more)								
Less than weekly	0.03	(0.02)	0.03	(0.02)	0.07*	(0.03)	0.07*	(0.03)
Co-residence	− 0.07	(0.09)	− 0.07	(0.09)	0.16	(0.10)	0.16	(0.10)
Transition to divorce * child at baseline								
Divorce * childless			0.23	(0.17)			− 0.31+	(0.16)
Divorce * child ≤ 14			0.15	(0.14)			− 0.17	(0.13)
Constant	10.86**	(1.66)	10.90**	(1.66)	12.33**	(1.57)	12.27**	(1.57)
R-squared	0.07		0.07		0.07		0.07	
No. of parents	22,093		22,093		23,570		23,570	
No. of dyads	28,495		28,495		30,603		30,603	
Observations	77,721		77,721		83,011		83,011	

Standard errors are clustered for parents. Control variables include dummies for waves, not reported in the table

**p < 0.01, *p < 0.05, +*p* < 0.1

### Differences Across European Contexts

In the third step of our analysis, we examine cross-context heterogeneity in the effect of children’s marital break-up. The association between children’s union dissolution and changes in the number of parents’ depressive symptoms was positive and significant within each of the European clusters, with the relevant exception of the group of Nordic European countries (Table 4). In Continental, Southern and Eastern Europe, older parents’ depressive symptoms increased when one of their children became divorced. The magnitude of the coefficients suggests that increases in parents’ depressive symptoms were the highest in Southern Europe (Coef. = 0.24), followed by Eastern (Coef. = 0.20), Continental (Coef. = 0.13) and Nordic (Coef. = 0.03) European societies. Between-clusters differences were further explored by fitting the regression model on the overall sample and including interaction terms between children’s divorce/separation and country groups in the analysis. The results from this latter analysis indicate that there was a statistically significant difference between Nordic and Southern European countries, and a marginally significant difference between Nordic and Continental Europe. In particular, the increase in depressive symptoms associated with family break-up was smaller for parents living in Nordic countries than for those living in Continental and Southern Europe. Conversely, there was no statistically significant difference between the effect observed in Eastern and Nordic European countries, probably because of the lack of sufficient statistical power.^9^ These results, mirroring what has been shown by Kalmijn and De Graaf (2012) at the micro-level, partly support our third hypothesis and a normative perspective of children’s life course events, suggesting that family union dissolution has less negative and non-significant consequences for parents’ mental health in contexts where the event is less stigmatized.

**Table 4:** Fixed effects linear regression models on the number of parents’ depressive symptoms

	Nordic		Continental		Eastern		Southern		Overall	
	Coef.	SE	Coef.	SE	Coef.	SE	Coef.	SE	Coef.	SE
Children’s characteristics										
Transition to divorce	0.03	(0.06)	0.13*	(0.06)	0.20*	(0.09)	0.24*	(0.12)	− 0.01	(0.07)
Transition to divorce * Continental									0.15+	(0.09)
Transition to divorce * Southern									0.23*	(0.11)
Transition to divorce * Eastern									0.15	(0.11)
Childbirth	0.01	(0.06)	0.01	(0.05)	0.00	(0.14)	0.05	(0.09)	− 0.04	(0.03)
Employment status (*Ref.* employed)										
Unemployed	0.03	(0.08)	0.04	(0.06)	0.19*	(0.08)	0.11	(0.08)	0.14**	(0.03)
Student	− 0.05	(0.07)	0.10	(0.10)	0.45+	(0.23)	0.09	(0.33)	0.10+	(0.06)
Not in LM	0.08	(0.06)	0.05	(0.04)	− 0.01	(0.06)	− 0.03	(0.07)	0.05*	(0.02)
Parents’ characteristics										
Age	− 0.14**	(0.05)	− 0.17**	(0.04)	− 0.31**	(0.07)	− 0.34**	(0.07)	− 0.06**	(0.01)
Age^2^	0.00**	(0.00)	0.00**	(0.00)	0.00**	(0.00)	0.00**	(0.00)	0.00**	(0.00)
Marital status (*Ref.* married)										
Divorce or separated	− 0.11	(0.26)	− 0.35	(0.25)	0.34	(0.32)	0.06	(0.64)	− 0.05	(0.10)
Widowed	0.34*	(0.14)	0.48**	(0.13)	0.86**	(0.17)	0.72**	(0.18)	0.63**	(0.04)
Employment status (*Ref.* employed)										
Retired	− 0.08	(0.05)	− 0.02	(0.05)	0.01	(0.07)	− 0.04	(0.10)	− 0.13**	(0.02)
Unemployed/not in LM	0.09	(0.07)	0.09	(0.06)	0.24*	(0.10)	0.06	(0.12)	0.07**	(0.03)
No. of chronic diseases	0.08**	(0.02)	0.08**	(0.01)	0.15**	(0.02)	0.15**	(0.02)	0.15**	(0.00)
No. of mobility limitations	0.14**	(0.02)	0.14**	(0.01)	0.16**	(0.01)	0.21**	(0.01)	0.17**	(0.01)
At least one limitation in IADLs	0.28**	(0.06)	0.28**	(0.04)	0.13*	(0.06)	0.51**	(0.07)	0.52**	(0.02)
At least one limitation in ADLs	0.29**	(0.08)	0.30**	(0.05)	0.45**	(0.08)	0.41**	(0.09)	0.59**	(0.02)
Parent–child contact frequency (*Ref.* weekly or more)										
Less than weekly	0.06	(0.04)	0.02	(0.03)	0.07	(0.04)	0.10	(0.07)	0.04*	(0.02)
Co-residence	0.28	(0.22)	0.07	(0.10)	0.06	(0.14)	− 0.13	(0.15)	0.07	(0.07)
Constant	4.98+	(2.56)	7.21**	(1.98)	15.94**	(3.21)	18.49**	(3.92)	5.62**	(0.80)
R-squared	0.05		0.06		0.07		0.11		0.05	
No. of parents	6763		14,892		7305		6724			
No. of dyads	11,667		24,514		11,514		11,399		59,094	
Observations	33,254		67,294		29,148		31,036		160,732	

Standard errors are clustered for parents. Control variables include dummies for waves, not reported in the table

**p < 0.01, *p < 0.05, +*p* < 0.1

## Discussion

Previous research has consistently documented that parental divorce has negative consequences on children’s life course outcomes (e.g. Amato and Cheadle 2008). However, far less attention has been paid to intergenerational effects in the opposite direction, i.e. from adult children’s partnership breakdown to older parents’ well-being. Two previous studies on the topic have reported mixed findings, showing a positive association between child’s family disruption on parents’ depressive symptoms in the Netherlands (Kalmijn and De Graaf 2012) and no effects in the USA (Milkie et al. 2008). The results presented in this study indicate that in the European context older parents’ depressive symptoms increased when one of their adult children divorced or separated. Adult children’s exposure to stressful life course events, such as marital break-up, reverberates into a decline in older parents’ mental health.

Our analyses also provide additional insights on the mediating and moderating factors affecting the association between child’s marital dissolution and changes in parent’s mental health. First, this association was not explained by changes in the frequency of intergenerational contact and co-residence. Older parents suffered from their children’s separation, regardless of the fact that intergenerational contacts increased or decreased following the event. Similarly, the effect of child’s divorce on parent’s depression symptoms was not mediated by the decision of parents or children to “double up” and live in the same household.

Second, contrary to our expectations, the association between child’s marital dissolution and parent’s mental health was neither exacerbated nor attenuated by the presence of a grandchild younger than 15 years. We found that while there were no differences between sons having young, old or no children, among childless daughters marital break-up was not associated with changes in parents’ depressive symptoms. The presence of (grand)children, of any age, seems to drive the negative association between daughter’s marital dissolution and parent’s mental health.

The results also provide partial support to our third hypothesis, based on a normative perspective of the life course: the negative consequences of family break-up vary across contexts and are smaller in those European contexts (i.e. Nordic Europe) in which divorce is more widespread and socially accepted. These findings add new evidence to the debate on the declining effect of divorce and suggest that social sanctions attached to family union dissolution may affect not only divorcees’ life courses, but also their parents’ mental health. In more traditional societies older parents may be judged for their socialization role when their children experience non-normative life transitions. This is in line with previous studies showing that parents in Southern European countries are particularly susceptible to their children’s family choices when are not accepted by the larger society (Di Giulio and Rosina 2007; Rosina and Fraboni 2004). Moreover, these parents may have more conservative values and interpret children’s divorce as an infringement of their beliefs. Our results could suggest that life course choices clashing with the values of parents have negative implications on parents’ mental health.

In interpreting these results a number of limitations should be considered. First, SHARE data do not include information about adult children’s well-being and depressive symptoms. We cannot exclude, therefore, that increases in parents’ depressive symptoms are consequences of children’s mental health problems following divorce. Second, children’s life course events may matter less for parents who have more than one child. A sensitivity analysis showed no interactions between a child’s marital break-up and sibling size, although many possible siblings’ characteristics could interact with a child’s divorce or separation. Third, parents’ mental health status may be susceptible to their children’s life course events when they are deeply involved in each other lives. Unfortunately, using SHARE data, it was not possible to analyse parent–child relationship quality prior to child’s divorce. In a sensitivity analysis, we used contact frequency at baseline as a proxy of relationship quality prior to marital break-up, and the results showed that parents in close contact with a specific child did not suffer more when he or she divorced than parents with less contact. This result is consistent with Pillemer et al.’s (2017) findings in the USA showing that parents’ psychological distress increased not only when the closest child had difficulties in his/her life, but also when any other child experienced some problems. Thus, the analysis presented in this paper assumes that there were no variations in the effects of children’s divorce according to parent–child closeness, but further studies on the topic are needed. A further limitation concerns unobserved characteristics of the child and his/her (ex-)partner. For example, the association between child’s divorce and the increase in the number of parent’s depressive symptoms may reflect extremely conflictive post-divorce relations between the ex-spouses. Highly conflictive post-divorce relations are more likely in Southern Europe where divorce takes place at higher levels of conflict (De Graaf and Kalmijn 2006; Härkönen and Dronkers 2006).

Despite these limitations, the empirical results presented here contribute to our knowledge of consequences of divorce and reveal that children’s life course events, such as union dissolution, can have important implications on older parents’ mental health. Although ageing process has led parents to enjoy sharing a longer part of their lives with children, the increasing complexity of family dynamics and the diffusion of non-traditional families in younger generations may have negative effects on the well-being of older generations. This provides further support to the claim that a “linked lives” approach is needed when studying the consequences of family dynamics on the well-being of older people. This study represents an example of how a multigenerational perspective can be combined with a longitudinal analysis and suggests a complex interplay between intergenerational linkages and non-normative life course transitions.

## Footnotes

1 Source: http://ec.europa.eu/eurostat/data/database; last accessed on May 31, 2018.

2 Jappens and Van Bavel (2012) used different indicators to synthesize family attitudes. They include: “When there are children in the home, parents should stay together even if they don’t get along”; “A woman should be prepared to cut down on her paid work for the sake of her family”.

3 The initial household response rate was equal to 61% in wave 1 (Börsch-Supan et al. 2013), and only 53% of these respondents were also present in wave 6. The attrition rate was 29% between waves 1 and 2, 33% between waves 2 and 4, 21% between waves 4 and 5, and 20% between waves 5 and 6.

4 Since the unit of analysis adopted is the parent–child dyad, parents with multiple children were present in multiple observations in the sample. Clustered standard errors were used to adjust the estimates for the correlation between dyads within the same parent.

5 Unfortunately, the SHARE data do not allow to know whether the young grandchild was born from the dissolved couple or from a previous relationship. However, it is very likely that most adult children did not experience two marital break-ups in few consecutive years.

6 A Wald test used to compare coefficients within the same model shows that the effects of widowhood and having limitation in daily activities were significantly larger than the one of children’s divorce (sign. = 0.000).

7 In an additional analysis, we added parent–child contact frequency and intergenerational co-residence in a series of subsequent nested models: the coefficients related to children’s divorce or separation did not differ substantially while including or excluding these variables from the regression model. We also examined whether the association between children’s transition to divorce and changes in parents’ depressive symptoms varied by parent–child contact frequency. The results show non-significant interaction terms between contact frequency and children’s union dissolution. These analyses are available from the authors upon request.

8 We performed additional analyses (available upon request) to understand whether the effect of children’s transition to divorce varied across father–son, father–daughter, mother–son and mother–daughter dyads. We did not find any significant interaction between children’s union dissolution and parent–child sex similarity.

9 When considering Southern European countries as the reference group, there were no significant differences between country groups, with the exception of Nordic Europe.
